# Attention and binding in visual working memory: Two forms of attention and two kinds of buffer storage

**DOI:** 10.3758/s13414-019-01837-x

**Published:** 2019-08-16

**Authors:** Graham J. Hitch, Richard J. Allen, Alan D. Baddeley

**Affiliations:** 1 University of York, York, UK; 2 University of Leeds, Leeds, UK

**Keywords:** Attention, Working memory, Visual working memory

## Abstract

We review our research on the episodic buffer in the multicomponent model of working memory (Baddeley, 2000), making explicit the influence of Anne Treisman’s work on the way our research has developed. The crucial linking theme concerns binding, whereby the individual features of an episode are combined as integrated representations. We summarize a series of experiments on visual working memory that investigated the retention of feature bindings and individual features. The effects of cognitive load, perceptual distraction, prioritization, serial position, and their interactions form a coherent pattern. We interpret our findings as demonstrating contrasting roles of externally driven and internally driven attentional processes, as well as a distinction between visual buffer storage and the focus of attention. Our account has strong links with Treisman’s concept of focused attention and aligns with a number of contemporary approaches to visual working memory.

Over 60 years have passed since Broadbent (1958) first presented a model linking short-term memory and attention. However, early investigation tended to concentrate on the auditory-verbal domain whereas in the visual domain, attention and short-term memory were typically studied separately. One development in bringing these perspectives together was the use of perceptually based change detection methods to investigate the capacity of visual working memory (Luck & Vogel, 1997). A parallel development was growing awareness of working memory among researchers studying attention in visual perception (e.g., Awh & Jonides, 2001; Downing, 2000; Lavie, 2005; Olivers, Peters, Houtkamp, & Roelfsema, 2011; Soto, Heinke, Humphreys, & Blanco, 2005). The latter included Treisman herself, especially in her later work (Wheeler & Treisman, 2002). In this article we review our research on attention and the episodic buffer in the revised multicomponent model of working memory (Baddeley, 2000), and show the important influence of Treisman’s original ideas on attention. The crucial linking theme concerns binding, whereby the component features of a stimulus are conjoined in integrated episodic representations in working memory.

We begin by briefly describing Treisman’s view that focused attention is necessary for encoding integrated representations of multifeatured objects in perception. We follow this with a summary of Luck and Vogel’s (1997) work and Wheeler and Treisman’s (2002) evidence that focused attention is involved in maintaining object information in visual working memory. We then introduce our own approach, which initially saw the storage of feature bindings as critically dependent on the limited-capacity resources of the central executive in the multicomponent model. As our work progressed we found it useful to make a broad distinction between internally driven attentional processes controlled by the central executive and externally driven processes of attending to external stimuli, as have many others (e.g., Egeth & Yantis, 1997; Chun, Golomb, & Turk-Browne, 2011; Lavie, Hirst, de Fockert, & Viding, 2004; Yantis, 2000). We go on to summarize a series of experiments that provide evidence on the contrasting roles of these two aspects of attention in visual working memory and, additionally, the need to distinguish between the current focus of attention and visuospatial buffer storage.

## Feature integration theory

Treisman’s major contribution was to demonstrate the importance of distinguishing between early and later stages of processing in visual perception. In early processing, different types of visual feature such as color and shape are analyzed separately in parallel streams. In later processing, the various features at an attended location are bound together, leading to the conscious percept of an integrated multifeatured object. The empirical evidence for this was based principally on perceptual tasks of figure–ground segregation and visual search (Treisman & Gelade, 1980). Thus, segregation of figure and ground is fast and easy when they are differentiated by a single salient feature, such as color or shape, but slow and effortful when the differentiation is marked by a conjunction of color and shape, such as a blue square among red squares and blue triangles. Similarly, visual search for a target stimulus is rapid and parallel when distractors differ from the target by a single feature but slow and serial when target and distractors share features and differ only by the way they are combined. Treisman was careful to point out that the question of what constitutes features and conjunctions is an empirical issue, a view upheld by Wolfe and Horowitz (2017), who have identified features as those stimulus characteristics that guide attention in a bottom-up manner, leading to rapid target detection. This approach has led to a modification of Treisman’s account, in which the initial parallel stage of perceptual processing involves a combination of top-down and bottom-up influences (Cave & Wolfe, 1990; Wolfe, Cave, & Franzel, 1989).

Our concern here, however, is with Treisman’s conceptual framework, feature integration theory (Treisman, 1986; Treisman & Gelade, 1980), which many years on remains highly influential (Humphreys, 2016). According to feature integration theory, the first stage of perception consists of pre-attentive processing. This generates a set of feature maps of their spatial distributions. The second stage consists of focused attention, which binds together information from a particular location in the various feature maps and leads to the perception of a multifeatured object at the location in question. In later work, Kahneman, Treisman, and Gibbs (1992) extended the theory to memory by developing the concept of a multifeatured object file as a temporary episodic representation. However, their main concern was with the role of object files in perception, whereas our own investigation of binding sprang from our interest in working memory and cognition more generally.

## Feature binding in visual working memory

Our recent work was partly stimulated by Luck and Vogel’s (1997) investigation of the storage capacity of visual working memory. Observers viewed a sample display of multifeatured objects, followed 900 ms later by a test display, the observers’ task being to decide whether the test display was the same or differed in some respect. Accuracy in this change detection task dropped as set size was increased beyond three or four objects but was unaffected by their visual complexity in terms of number of features. Luck and Vogel took this to imply that visual working memory is an object-based store with a fixed capacity limited to three or four objects. Their findings led to a surge in interest and further experimentation, including challenges to the claim that features do not take up capacity (e.g., Alvarez & Cavanagh, 2004; Oberauer & Eichenberger, 2013) and evidence for more flexible resource allocation models of storage capacity (e.g., Bays & Husain, 2008). Notwithstanding these issues, the important implication of Luck and Vogel’s results for our present purposes is that the capacity to store object information in visual working memory clearly exceeds the capacity of focused perceptual attention. Thus, whereas only one object file can be active at any time in perception, a larger yet limited amount of information can be represented simultaneously in store. As we discuss in more detail later, this disparity underlines the importance of a fundamental distinction between two different types of capacity in working memory, one concerned with storage and the other with perceptual attention.

Treisman’s own investigations of visual working memory were based on her observation that Luck and Vogel’s (1997) experiments could be performed by remembering individual features without reference to feature bindings. Together with Wheeler, she devised conditions that allowed memory for features and feature bindings to be compared, finding that the outcome depended critically on whether change detection was performed on a whole array or a single item (Wheeler & Treisman, 2002). Thus, when the change detection stimulus was a whole array, bindings were remembered less well than individual features, but when it was a single item this difference disappeared. Wheeler and Treisman argued that the requirement to search a whole array would place high demands on perceptual attention and interpreted their results as suggesting that focused attention is involved in maintaining bound object representations in working memory.

## Binding in the multicomponent model of working memory

**Fig. 1:**
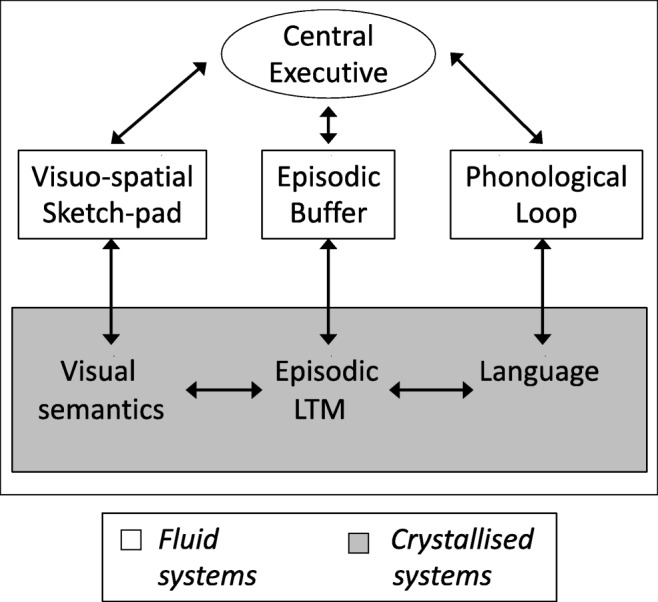
The revised multicomponent model of working memory. From “The Episodic Buffer: A New Component of Working Memory?” by A. D. Baddeley, 2000, *Trends in Cognitive Sciences*, *4*, p. 421. Copyright 2000 by Elsevier Inc. Adapted with permission

At about the same time, we ourselves were exploring the hypothesis that attention plays a critical role in encoding and maintaining bound representations in working memory. However, in our case the question referred to internally directed attention controlled by the central executive and was asked in the context of binding more generally. The background was an extension of the original multicomponent model of Baddeley & Hitch (1974) to account for the temporary storage of integrated episodic representations (Baddeley, 2000). These include object representations in the visual domain but extend to chunking involving other subsystems in working memory as well as long-term memory. The revised model addressed the problem of binding, both within and between subsystems, by proposing an episodic buffer, a limited-capacity multimodal store specialized for holding integrated representations (see Fig. 1). The episodic buffer was assumed to be closely linked to the central executive to capture the idea that its contents are available to consciousness. Our investigations have included chunking in the verbal domain (Baddeley, Hitch, & Allen, 2009); however, the present discussion concentrates on feature binding in the visual domain as this has told us a good deal about the roles of different types of attentional process in working memory. We began by exploring the assumption that forming bound representations involves the central executive.

## Preview of methods

We began by using change detection to compare memory for feature bindings and individual features in sets of three or four colored shapes. In later work we used cued recall to study memory for bindings in more detail. Our methods were initially based on ones we had used previously to investigate working memory—in particular, the dual-task methodology to examine the effect of cognitive load. A second method was to present stimuli sequentially, as is more typical of experiments on verbal working memory. We used simultaneous presentation also, finding that the two presentation methods give similar results. However, the advantage of being able to study memory for objects as a function of their recency of presentation turned out to be particularly informative.

By way of overview, Table 1 lists our main experimental manipulations, together with our assumptions about the underlying basis of their effects. In dual-task studies, a concurrent activity involving high or low cognitive load was required during the visual working memory task. High cognitive load was typically achieved by counting backward from a multidigit number. Low cognitive load consisted of articulatory suppression, typically the repetition of a single multidigit number. Both tasks involve similar verbal output and therefore reduce the opportunity to use verbal recoding to similar extents, with the critical difference being that counting places a substantially higher load on the central executive. Thus, if concurrent counting impaired one aspect of memory performance while sparing another, we concluded that the aspect in question drew more heavily on the limited-capacity resources of the central executive.

**Table 1:** Summary of the principal experimental manipulations and their assumed locus of effect in working memory

Method	Description	Assumed Basis of Effect	Examples
Dual-task interference	Requirement to count backward during the visual memory task	Takes up internal attentional capacity (central executive)	Allen, Baddeley, & Hitch (2006) Allen, Hitch, Mate, & Baddeley (2012)
Perceptual distractor (Stimulus suffix)	Presentation of a visual distractor in the retention interval	Draws the focus of perceptual attention to the distractor	Ueno, Allen, Baddeley, Hitch, & Saito (2011a) Hu, Hitch, Baddeley, Zhang, & Allen (2014)
Prioritization instructions	Instructions that correct recall earns a different number of reward points for different items	Alters the deployment of internal attentional capacity (central executive)	Hu, et al. (2014) Hitch, Hu, Allen, & Baddeley (2018)

Our second manipulation involved presenting an additional colored shape in the short retention interval between study and test. Participants were instructed to ignore this perceptual distractor, which we refer to as a “stimulus suffix,” the term used for a similar manipulation in the auditory–verbal domain. In verbal short-term memory an auditory stimulus suffix causes selective interference with the ability to recall the most recently presented item (Crowder & Morton, 1969), but little is known about any potential equivalent in visual short-term memory. Our experiments revealed a somewhat analogous effect. As we go on to explain, we assume this is because a suffix tends to draw perceptual attention and gain access to visual working memory, where it interferes with representations in store. The interesting finding was that the interference caused by a visual suffix was selective, suggesting distinct forms of storage within visual working memory.

Our third variable was prioritization. The need to investigate this arose from an experiment in which we observed some participants concentrating on remembering just one or two items in a short series. We interpreted this as a strategic response to the difficulty of trying to remember all the items. Given that implementing strategies is a function of the central executive, we investigated further. We did so by instructing participants they would obtain different numbers of notional “points” for correctly remembering items according to their position in the sequence. We interpreted the effects of such prioritization instructions to reflect differences in strategies for allocating central executive resources in the memory task.

To summarize, we used dual-task interference and prioritization instructions as our principal tools for investigating the role of the central executive, and the presentation of a stimulus suffix to investigate the role of perceptual attention. By combining these manipulations and studying their interactions with serial position, we sought to shed light on the way internal and external attention combine to influence visual working memory.

## Preview of findings

In the most general terms, our results suggest that external–perceptual and internal–executive attention interact with different forms of buffer storage in visual working memory. More specifically, we distinguish between the visuospatial sketchpad and the current focus of attention. Our findings suggest that attending to an external stimulus creates an object file in the focus of attention that remains until displaced by a subsequent stimulus. In contrast, the visuospatial sketchpad is capable of holding partial information about a number of object files as they undergo fragmentation in store. Executive processes are responsible for strategies for using these resources to satisfy the current goals. One example is “attentional refreshing,” a control process of reactivating pieces of stored information one at a time (Barrouillet & Camos, 2014). Our studies suggest that schedules of attentional refreshing vary with the task goals, as for example when instructions are used to assign higher importance to some of the information in visual working memory.

## Dual-task studies

We began by exploring whether the binding processes underpinning integrated representations in working memory are crucially dependent on the central executive. If so, loading executive resources with a demanding current task would be especially damaging to memory for feature bindings. However, in a series of dual-task studies, the cognitive load of backward counting impaired memory for bindings no more than memory for individual features (Allen, Baddeley, & Hitch, 2006; Allen, Hitch, Mate, & Baddeley, 2012).^1^ This was the case regardless of whether the study sample of colored shapes was presented simultaneously or sequentially (Allen, Baddeley, & Hitch, 2014). It was also the case when binding was made more demanding by requiring the integration of features over a spatial or temporal interval or even across modalities, for example by presenting shapes visually and colors auditorily (Allen, Hitch, & Baddeley, 2009; Karlsen et al., 2010). In light of these consistently negative results, we abandoned our initial hypothesis, concluding that the episodic buffer is a passive system for combining information from a range of dimensions and sources and making it available to conscious awareness but does not itself serve a binding function (see Baddeley, 2012; Baddeley, Allen, & Hitch, 2011).

**Fig. 2:**
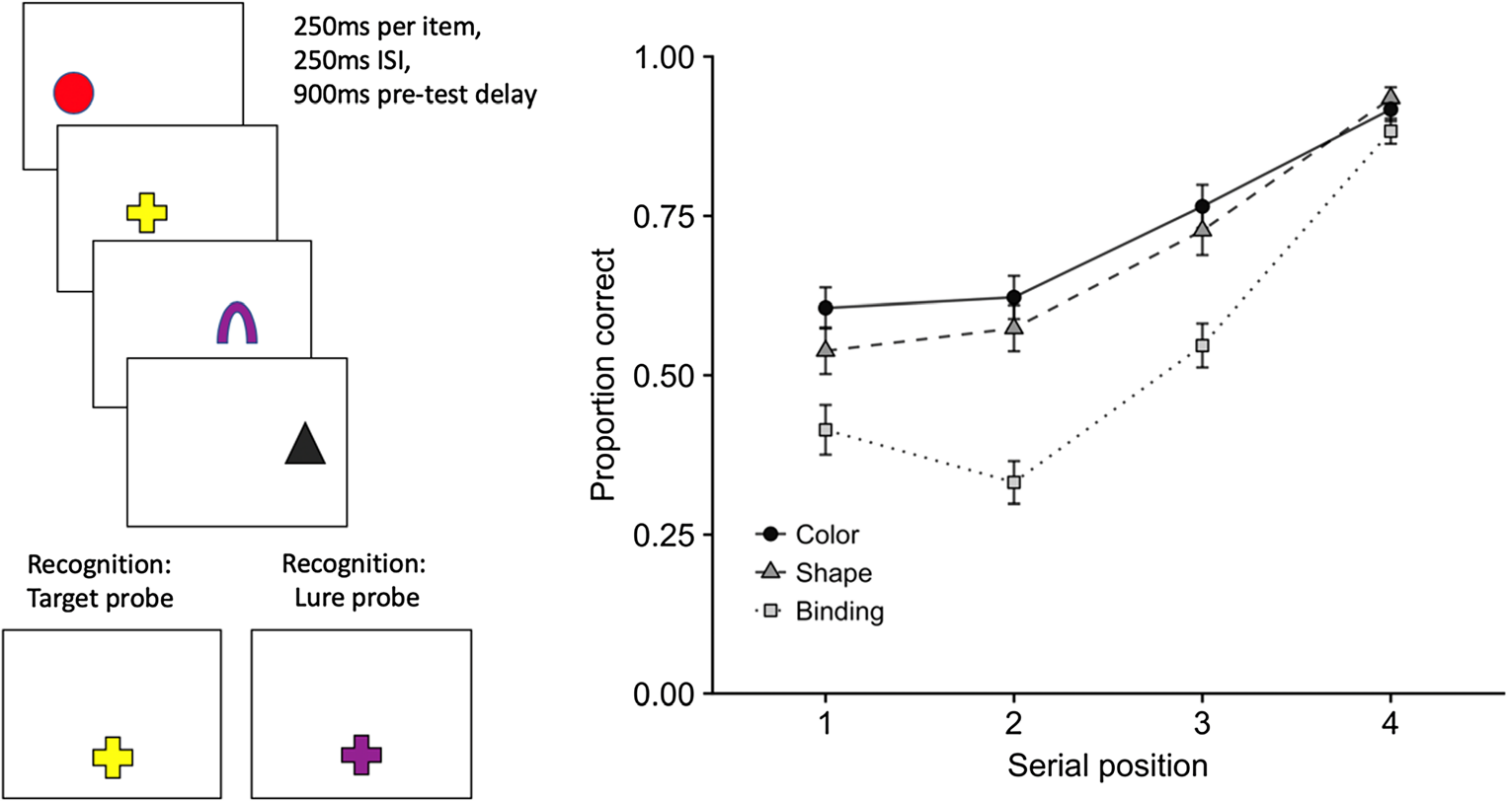
Schematic of the methodology (illustrating the binding condition) and proportions correct on probe-present trials in different stimulus conditions as a function of serial position (Allen et al., 2006, Exp. 5). The presentation and test phases in the color and shape conditions involved colored squares and unfilled shape outlines, respectively

An insight into the way visual feature bindings are maintained came from an experiment in which colored shapes were presented sequentially and memory was tested by single-probe recognition (Allen et al., 2006). There was a clear recency effect in all conditions, with the last-presented item remembered best, as is typical for visual stimuli (Kerr, Avons, & Ward, 1999; Phillips & Christie, 1977; Walker, Hitch, & Duroe, 1993). This appears to be due to later items interfering with items already in the store (Kool, Conway, & Turk-Browne, 2014). The new finding was that recency was more pronounced for shape–color bindings than for shape or color in isolation (see Fig. 2; see also Brown & Brockmole, 2010; Brown, Niven, Logie, Rhodes, & Allen, 2017). The greater fragility of bindings would follow if they were represented with less redundancy than features (Treisman & Zhang, 2006)—for example, if forgetting involves the fragmentation of integrated representations (cf. Jones, 1976), such that bindings fall apart before features.

The conclusion that representations of feature bindings are fragile can be related to Wheeler and Treisman’s (2002) suggestion that maintaining integrated representations requires focused attention. On the one hand, the completeness of memory for the final item can be taken to reflect access to the most recent object file associated with focused attention (see Walker, Hitch, Doyle, & Porter, 1994; see also Kahneman et al., 1992). On the other hand, the faster forgetting of bindings for older items can be attributed to the fragmentation of representations within the memory store. This account embodies the distinction noted earlier between the smaller capacity of focused attention and the larger but still limited storage capacity of visual working memory.

**Fig. 3:**
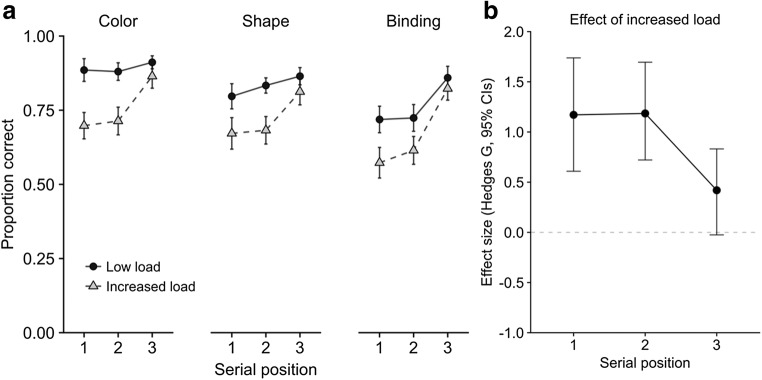
(a) Serial position curves showing single-item change detection accuracy for color, shape, and binding as a function of concurrent task load (Allen et al., 2014, Exp. 2), and (b) the mean effect sizes of increased load at each serial position across eight experiments. Effect size was calculated using the bootES package (Kirby & Gerlanc, 2013)

Converging evidence for distinguishing these two components of storage in visual working memory came from dual-task experiments exploring the effect of concurrent counting when study items were presented serially (Allen, Baddeley, & Hitch, 2014). Cognitive load disrupted memory for the features and bindings of earlier items, with minimal impact on the most recent one, consistent with this item having a different status in working memory (see Fig. 3a). Indeed, this basic pattern of findings appears highly consistent across a range of studies. To demonstrate this, we calculated the mean concurrent-task effect sizes in eight experiments (*N* = 18–26 per experiment, 184 in total) carried out by our group, employing manipulations of executive load on visual working memory using three-item sequences (Allen et al., 2009, Exp. 3; Karlsen et al., 2010, Exp. 2; Allen et al., 2014, Exps. 1–3, plus an additional unpublished experiment closely based on Exp. 1 of that series; Allen, Baddeley, & Hitch, 2017, Exps. 6–7). As is illustrated in Fig. 3b, this effect was substantially larger for early positions, relative to the final position. Finally, convergent evidence can also be drawn from an individual differences approach exploring memory for sequences of colored shapes in children 7–10 years of age. Berry, Waterman, Baddeley, Hitch, and Allen (2018) found that memory for the first two items in a three-item sequence correlated with performance on broader measures of verbal and visuospatial working memory, but no such relationship was apparent for the final sequence item.

We assume that, when available, executive resources are devoted to internal attentional refreshing, which reactivates the traces of items undergoing forgetting, in the manner suggested by Barrouillet and Camos (2014). Refreshing would strengthen either features or bindings depending on how much forgetting has taken place. We assume there is no need to refresh the object file of the most recently presented item, as this is still intact and is accessed automatically on presentation of the change detection probe.

## Stimulus suffix effects

**Fig. 4:**
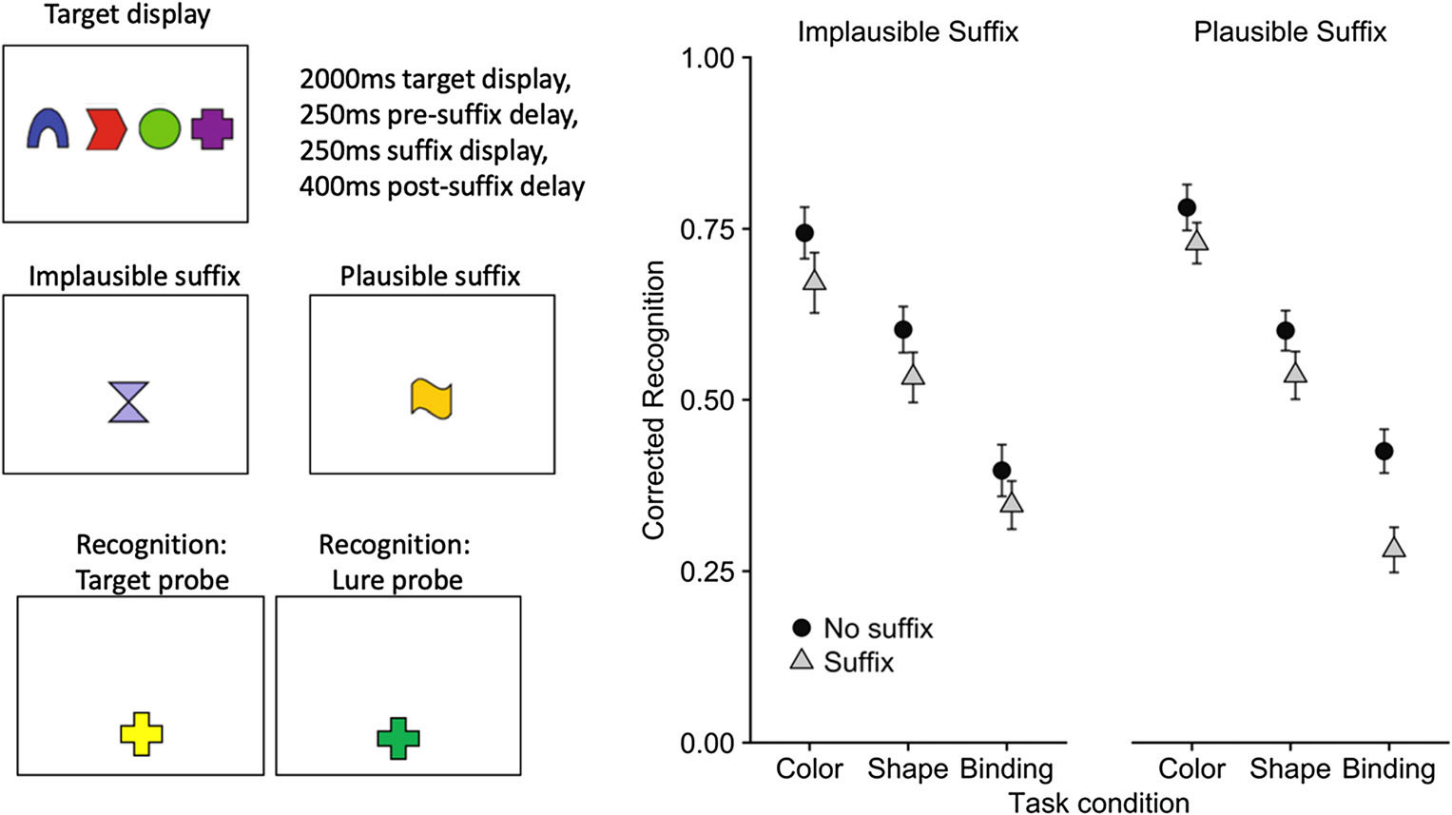
Schematic illustration of the methodology (see Ueno, Allen, et al., 2011, for full details), and effects of two types of suffix distractor on single-probe change detection for features and bindings (Ueno, Allen, et al., 2011, Exps. 2 and 3a)

Further evidence for the distinctive status of recently presented information in visual working memory comes from the effect of a stimulus suffix distractor in the interval between study and test. Initial experiments established that a stimulus suffix interferes with memory to an extent that depends critically on its visual features (Ueno, Allen, Baddeley, Hitch, & Saito, 2011a). These experiments studied memory for a simultaneously presented array of colored shapes. Two kinds of suffix were presented. A plausible suffix was one whose color and shape were drawn from the pool used to generate the study items, but not used on that trial. In contrast, the shape and color of an implausible suffix were drawn from a distinctly different set from the study items. Thus, a plausible suffix could readily be mistaken for a study item, whereas this was not the case for an implausible suffix. An implausible suffix had the same negative impact on memory for features and bindings, whereas a plausible suffix had a greater impact on bindings (see Fig. 4). Subsequent experiments explored further using cued recall, in which the test probe was the color or shape of a study item and participants had to recall its missing feature (Ueno, Mate, Allen, Hitch, & Baddeley, 2011b). Cued recall is a more sensitive test of memory for feature bindings and gives extra information in the form of errors. The results showed that a plausible suffix led to misbinding errors of recalling the color or shape of the suffix. We assume that participants have difficulty ignoring a suffix, especially one with plausible features, and that when the suffix is attended it gains access to visual working memory, where it causes interference with information already in store.

**Fig. 5:**
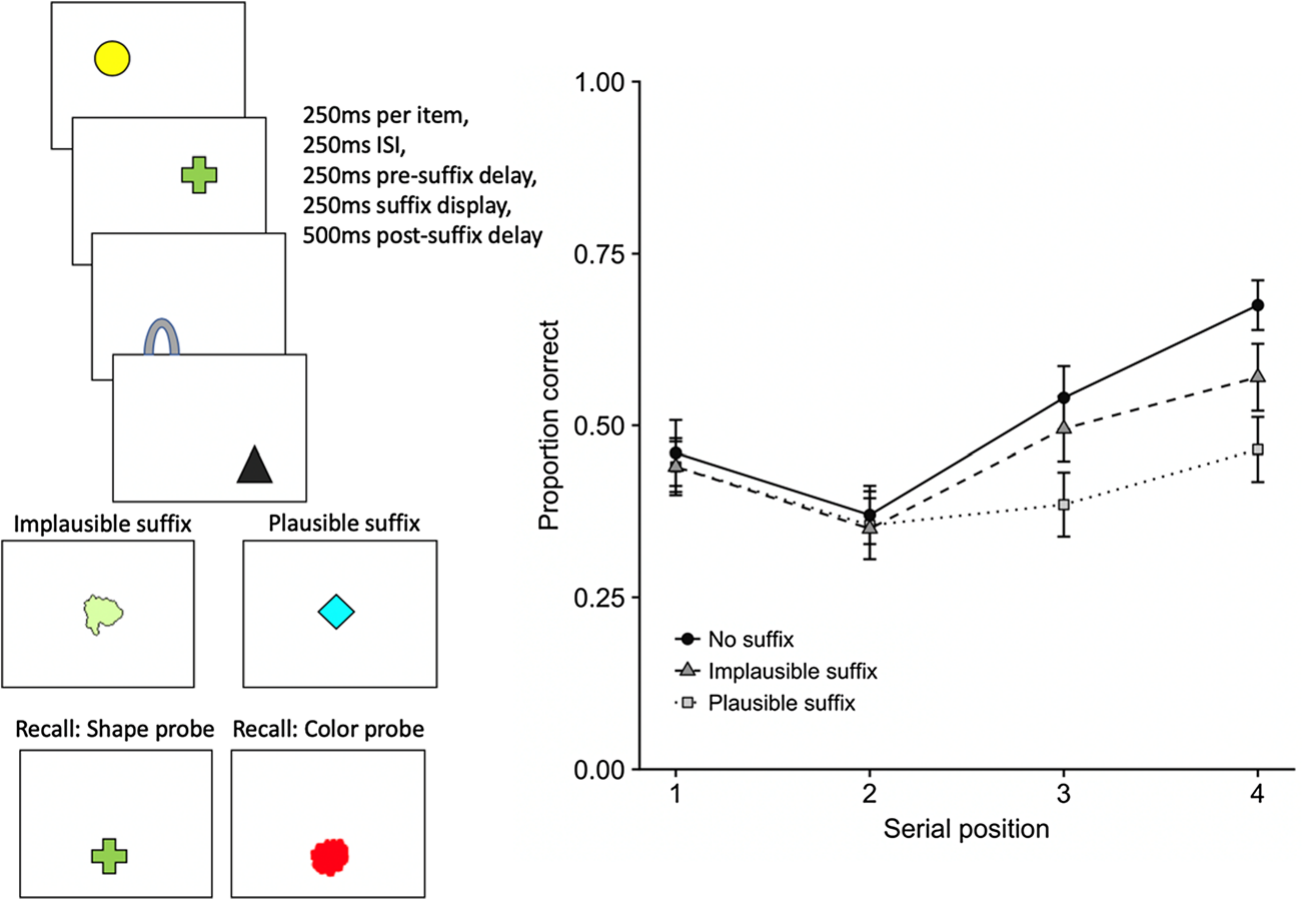
Schematic of the methodology, and proportions of correct cued recall as a function of the item’s serial position (Hu et al., 2014, Exp. 1)

Evidence linking suffix interference to recency emerged from further experiments using sequential presentation and cued recall (Hu, Hitch, Baddeley, Zhang, & Allen, 2014). We predicted that an inadvertently attended stimulus suffix would cause greatest impairment to memory for the final study item by supplanting its status as the most recent stimulus. The results confirmed this and replicated the plausibility effect at the same time (see Fig. 5). Memory for the penultimate item was also affected, though less so, suggesting some kind of knock-on effect when the final item is displaced. Setting this detail aside for the moment, the results suggest a double dissociation, whereby recent but not early items are sensitive to a stimulus suffix, whereas early items but not the most recent are sensitive to cognitive load (cf. Figs. 3 and 5).

## A preliminary interpretation

Thus far, we have described the effects of serial position, dual-task interference, and a suffix distractor on visual working memory. The ways these variables interact seem to converge on a relatively simple account in which the capacity for storage exceeds the capacity of focused attention and in which perceptual attention and internal attention perform fundamentally different roles. Put simply, perceptual attention acts as a gateway to visual working memory and leads to the initial creation of an integrated object file, whereas executive control is important for maintaining object files once they start to undergo interference in store. The most recent object file appears to have a different status from the representations of older items, as shown by excellent memory for the last presented item and its susceptibility to interference from a suffix. Memory for binding information is particularly fragile, possibly reflecting the fragmentation of object files in store. The executive processes used to offset forgetting seem to involve reactivating object files through attentional refreshing, which strengthens bindings or individual features depending on the extent of fragmentation. In summary, therefore, we envisage a visual working memory system in which each cycle of perceptual attention creates a new object file that momentarily has a special status, being complete and highly accessible. Each such cycle can be thought of as pushing back the object files of immediately previous stimuli in store, resulting in a recency gradient in which binding information is lost faster than feature information.

In all the experiments so far, each study item had the same importance for retention and we assume this will have influenced the way executive resources were allocated. Modifying the task goals should therefore alter the way the executive is set up to operate. To investigate this possibility, we explored the effects of assigning different priorities to different study items. In general, we expected more resources to be allocated to high priority items and for their recall to improve as a result. On the other hand, given the limited capacity of the central executive, memory for low-priority items should drop. We assumed further that examining this trade-off when items were presented sequentially might shed further light on the question of the special status of the most recent object file.

## Prioritization effects

**Fig. 6:**
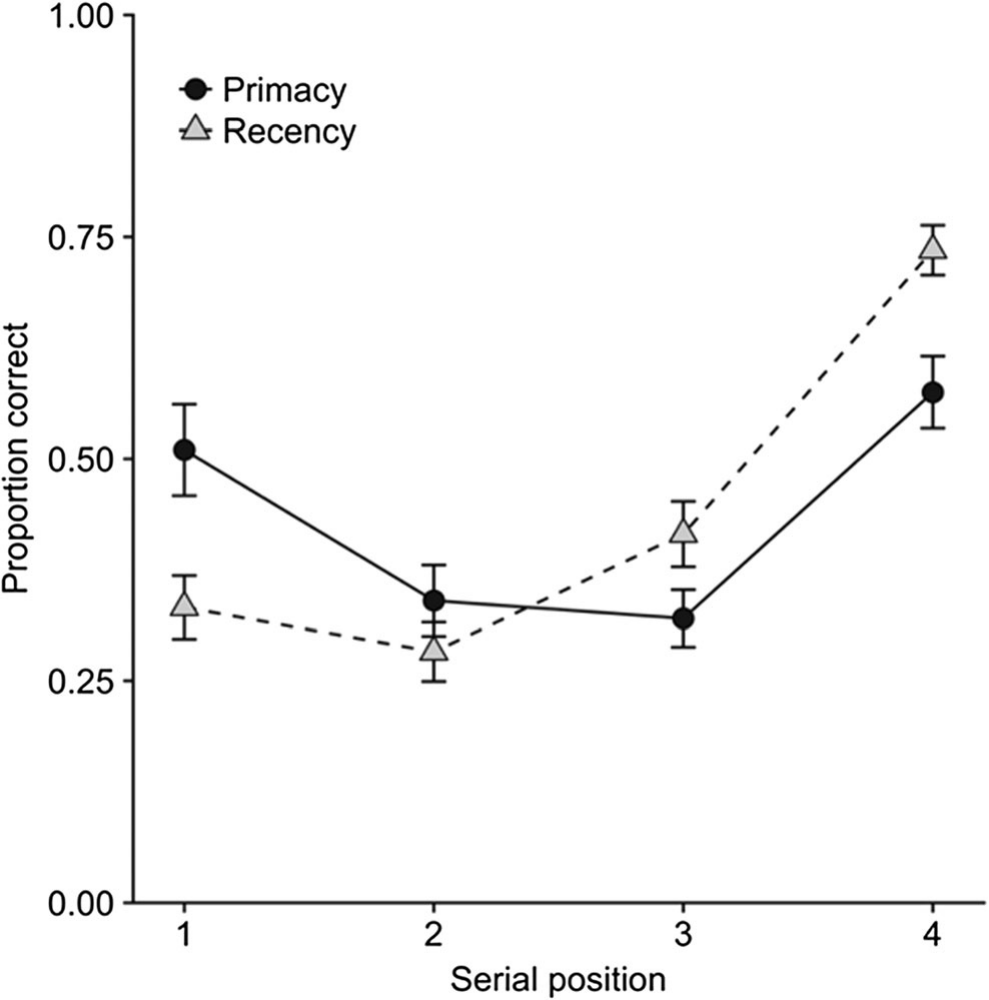
Effects of different prioritization instructions on serial position curves in cued recall (Hu et al., 2014, Exp. 4)

**Fig. 7:**
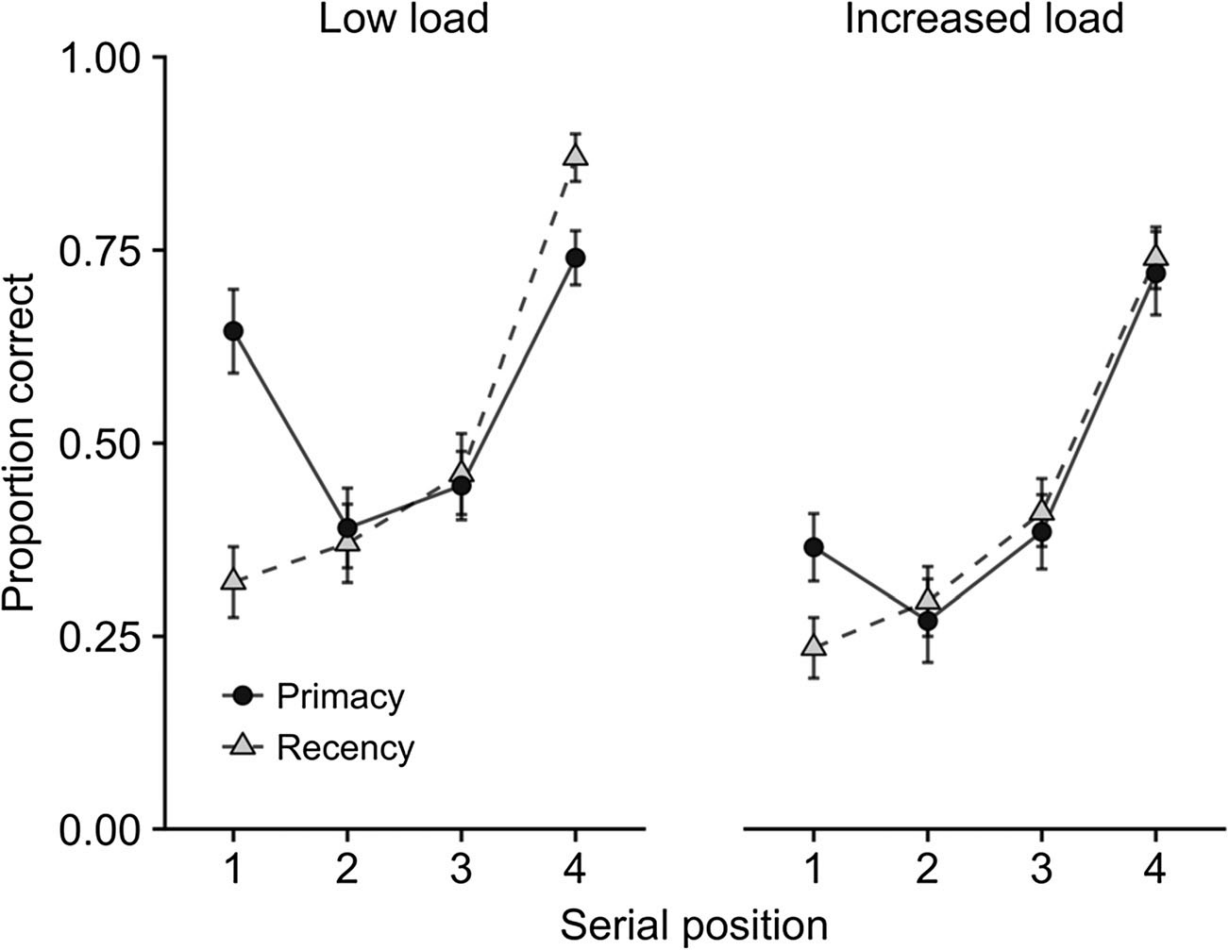
Effects of prioritization instructions on cued recall of a single item from a series of four colored shapes with articulatory suppression (low load) and with concurrent counting (high load) (Hu et al., 2016)

We continued to examine memory for bindings using cued recall in which the shape or color of one of the study items served as the cue for recall of its other feature. As we described above, prioritization was manipulated by awarding different numbers of points for the correct recall of items from different serial positions. One experiment compared prioritization schemes that emphasized either primacy, by giving most points for recall of the first item, or recency, by giving most points for the last (Hu et al., 2014, Exp. 4). As expected, gains in recalling high-priority items were offset by poorer memory for low-priority items, such that the overall amount of information recalled was the same (see Fig. 6). Further experiments revealed similar trade-offs for different reward schemes and showed that these were relative to a baseline in which all items were assigned the same priority (Hitch, Hu, Allen, & Baddeley, 2018). Dual-task studies confirmed that these trade-offs were mediated by the central executive, as they were considerably reduced under the cognitive load of concurrent counting (Hu, Allen, Baddeley, & Hitch, 2016). Figure 7 illustrates the effect of cognitive load on the comparison between instructions emphasizing primacy or recency. We have also observed that children 7–10 years of age are able to prioritize within visual working memory, at least when sufficiently motivated to do so, though such effects appear to be somewhat smaller than those observed in adults (Atkinson, Waterman, & Allen, 2019).

An interesting further feature of the results is that the most recent item was always remembered best, regardless of prioritization. This is consistent with the hypothesis that the last item has a special status in working memory that does not draw noticeably on executive resources. This hypothesis predicts that a stimulus suffix will disrupt memory for the most recent item, independently of which items are prioritized. To explore, we compared the effect of presenting a suffix when prioritization instructions emphasized either recency or primacy (Hu et al., 2014).

**Fig. 8:**
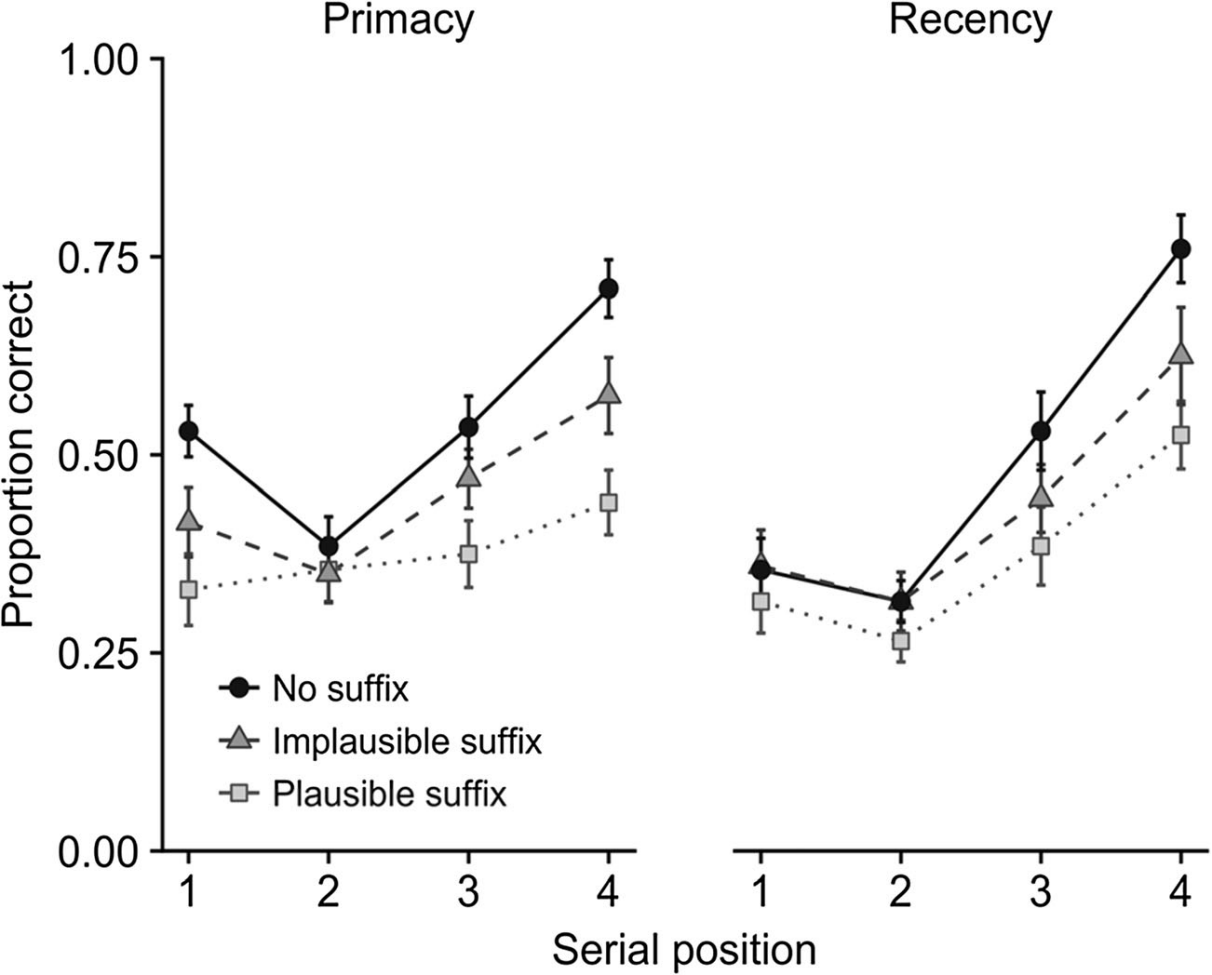
Effects of a plausible or an implausible suffix on cued recall of an item as a function of its serial position under prioritization conditions emphasizing primacy or recency (Hu et al., 2014)

Our expectation was confirmed, in that in both prioritization conditions, memory for the most recent item was impaired by a stimulus suffix, with a bigger effect when the suffix was plausible (see Fig. 8). These effects extended to the penultimate item, as we had found previously with no prioritization instructions. There was, however, an unexpected finding, in that when primacy was prioritized, presentation of a suffix also reduced the boost to memory for the first item, and did so to a greater extent when its features were plausible. Taken together, these results suggest that the form of representation of the most recent items can also underpin memory for the first item, given appropriate prioritization instructions. Further experiments have shown that this result generalizes, in that the same combination of enhanced recall and increased vulnerability to suffix interference appears for any item given high priority (Hitch et al., 2018), with these findings also extending to simultaneous presentation of multi-item arrays (Allen & Ueno, 2018). It seems, therefore, that internally directed attention can be used to maintain memory representations in the state they occupy automatically upon receiving perceptual attention. We think of this as a privileged state within visual working memory, characterized by heightened accessibility but increased vulnerability to perceptual interference, broadly equivalent to the focus of attention identified by others (Cowan, 2011; Oberauer & Hein, 2012; Souza & Oberauer, 2016) and closely related to Treisman’s concept of focused attention in perception.

Our earlier findings suggested that in the absence of prioritization instructions, executive processes are used to offset the forgetting of older items through the attentional refreshing of features or bindings. The question arises of why attentional refreshing does not render older items sensitive to interference from a suffix, whereas maintaining a prioritized item does. At present, we do not have the answer, but one interesting possibility is that prioritization increases the probability of a particular item being refreshed and decreases the probability of refreshing representations relating to other items. Thus, on a proportion of trials, an integrated representation of an important item could be actively maintained right through from initial perception to the moment of test. If this has the effect of preserving the item’s recency status, it would explain the increase in susceptibility to interference from a stimulus suffix. Our dual-task experiments suggest that the poorer retention of less important items results from the limit on executive resources. However, to the extent that attentional refreshing involves the focus of attention, it may also reflect the limited representational capacity of this key component of working memory.

Thus, to summarize a somewhat complex set of results, we find that prioritizing items in visual working memory boosts their retention, at the expense of poorer recall of other items. When the changes in probability of recall are aggregated over all four serial positions, the amount of information entering into this trade-off approximates to a single item on any given trial (Hitch et al., 2018). Prioritization effects are reduced by a concurrent cognitive load, suggesting their dependence on limited-capacity resources. However, the boost in recall due to prioritization is also vulnerable to perceptually driven interference from a suffix distractor. Under all conditions of prioritization, the most recent items are remembered best and are vulnerable to suffix interference, while being unaffected by cognitive load.

## General discussion

We set out to explore binding in visual working memory using the revised multicomponent model of Baddeley (2000) as a framework and methods more familiar in the verbal domain, such as dual-task interference, sequential presentation, and stimulus suffix effects. This approach offers a somewhat different perspective from those of researchers with primary interests in visual perception and attention, both methodologically and in terms of theoretical framework. However, our overall conclusion is that these approaches are complementary and convergent, informing each other in useful ways. One example is our evidence that internal and external attention operate in different ways in working memory, fleshing out an earlier suggestion by Cowan (1988). Thus, research on perception has emphasized a specific role for selective attention in encoding bound representations, whereas our dual-task experiments demonstrated a more general and flexible role for internally directed attention in response to task goals. This is illustrated by the allocation of limited-capacity executive resources for attentional refreshing depending on how stored information is prioritized. Another example of complementarity and convergence is our evidence that recent information is stored differently from older information. It was already known from the work of Luck and Vogel (1997) that the storage capacity of working memory exceeds the single-item capacity of perceptual attention. Our use of sequential presentation helps interrelate these two capacities, by demonstrating a dissociation between memory for recent and older information, as, for example, in their differential sensitivities to dual-task and suffix interference. In general terms, therefore, a minimal account of visual working memory must at the very least address the different roles of external and internal attention and their relationship to the different ways that recent and older information are stored, broadly consistent with general conclusions based on a wider range of phenomena (e.g., Awh, Vogel, & Oh, 2006; Niklaus, Singmann, & Oberauer, 2019).

At several points we have attempted to specify how different aspects of attention and storage operate in more detail, using evidence from interactions and serial position curves. We should emphasize that the scope for these attempts has been limited by the information in these curves, as they aggregate data over trials on any of which only a single item is probed. This limitation is important in a number of respects. One concerns the question of precisely which items in a sequence qualify as recent. Thus, our dual-task studies showed that only the final item is relatively immune to interference from counting backward, whereas our studies of suffix interference typically showed an effect on the penultimate as well as final items. Given the limitations of our methodology, we are inclined not to be too concerned by this disparity, for the present. It nevertheless remains to be investigated and resolved. The aggregation of data in serial position curves is also important for interpreting the costs and benefits associated with different prioritization instructions. We noted that these trade off in such a way that the flexible component approximates to the retention of a single item. However, more powerful methods will be required in order to answer questions about how this comes about on individual trials, as well as our suggestion that attentional refreshing is utilized somewhat differently when some of the information in visual working memory is designated as having higher priority for recall.

Despite the limitations of our methods, we suggest they have provided fresh insights and clear support for a multicomponent account of visual working memory. To reiterate briefly, attending to a stimulus creates an object file that is accessible but at the same time vulnerable to interference when another stimulus is perceived. This can account for the observation that the last item in a sequence is typically the best remembered unless the last item is followed by a suffix distractor. As regards the retention of older items, we attribute the recency gradient over serial positions to the cumulation of retroactive interference as successive stimuli are encoded. Our assumption that interference involves the progressive fragmentation of object files provides a simple explanation for the faster forgetting of feature bindings than feature values. Our initial dual-task studies showed that executive resources can be used to reactivate the degrading representations of older objects, and that this can involve refreshing individual features as well as bindings, consistent with forgetting through fragmentation.

**Fig. 9:**
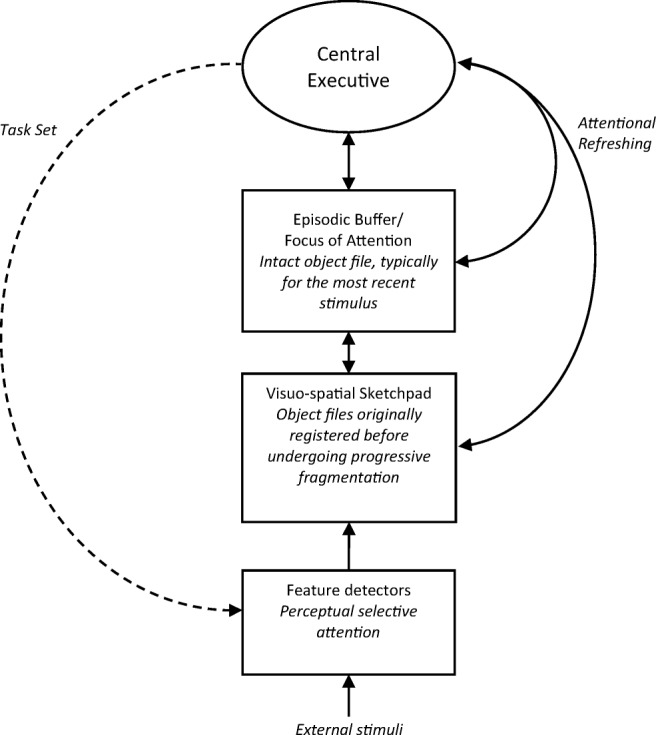
Diagram showing the principal components of visual working memory and their functions. Note that *task set* refers to the initial setting of perceptual filters to select task-relevant stimuli. Once these are set, stimulus selection is largely obligatory

Our experiments on prioritization effects were important in indicating that the partition of storage in visual working memory is not based solely on the distinction between recent and older information. The results suggested that prioritizing an older item increases the probability of its object file having the same status as a recently encoded object, being not only highly accessible but also vulnerable to perceptual interference from a suffix distractor. Our suggestion that this reflects maintaining a single item within the focus of attention bears an obvious similarity to Treisman’s concept of focused attention in perception. Given our present level of understanding, we regard the two concepts as broadly equivalent, with Treisman emphasizing attention as a process, and ourselves being concerned rather more with the memory representation created by attending to a stimulus. Figure 9 shows an expansion of a model we described earlier (Baddeley et al., 2011) that illustrates our current view of how the system operates.

The concept of the focus of attention as a subregion within working memory is a feature of a number of current models, but they differ as to its representational capacity. Thus, according to Cowan (2011), the focus of attention has a capacity of three or four chunks, whereas according to Oberauer and Hein (2012), it only holds a single item or chunk (though see Oberauer, 2018, for a recent adjustment to this view). Our data are quantitatively closer to the lower end of this capacity scale, though with some evidence that more than one item can be concurrently prioritized (Allen & Ueno, 2018; Hitch et al., 2018). In another respect, our theoretical position is radically different from both models, and from others like them that regard working memory as the currently activated region of long-term memory. In contrast, the multicomponent model assumes a set of short-term buffer stores. Baddeley, Hitch, and Allen (2019) summarized the principal arguments against viewing working memory solely in terms of activated long-term memory (see also Norris, 2017, and Cowan, 2019, for discussions). We note also that Engle’s (2018) account of working memory as a combination of executive attention with an information maintenance function can be regarded as a high-level simplification of the multicomponent model that lacks sufficient detail to account for the effects of different types of attention and of the partition of storage implied by the present results.

We should also point out that our evidence on the focus of attention is complementary to the large amount of evidence from studies of retro-cueing (see Souza & Oberauer, 2016, for a review). A retro-cue is presented after exposure to a study sample and informs participants which item (or items) in working memory are most likely to be tested for retention. The idea is that the prioritized information is brought into the focus of attention in response to the retro-cue. This contrasts with our procedure of prioritizing items in the study sample through instruction before they are presented. It also differs from our procedure in that the retro-cue typically provides information about the probabilities that different items will be tested whereas all items had an equal probability of being tested in our studies. Indeed, Atkinson et al. (2018) suggested that this may be an important methodological distinction, and that information regarding an item’s value and its probability of being tested may motivate distinct forms of attentional direction.

It is interesting to note that research on retro-cueing has led to a number of similar conclusions, notably concerning the existence of a trade-off whereby enhanced memory for high-priority information comes at the cost of poorer memory for low-priority information (Gunseli, van Moorselaar, Meeter, & Olivers, 2015). However, there are also some striking differences. A noteworthy example is evidence that postcueing appears to protect the selected representation from perceptual interference (Makovski & Jiang, 2007; Makovski, Sussman, & Jiang, 2008; Matsukura, Luck, & Vecera, 2007; Souza, Rerko, & Oberauer, 2016; van Moorselaar, Gunseli, Theeuwes, & Oliver, 2014), which is the opposite of what we find here. How can we account for this discrepancy? The answer may have to do with the fact that executive resources can be configured in different ways to respond to task demands, as we have already suggested. Thus with a retro-cue attentional refreshing can only begin after encoding and may be coupled with some form of consolidation. However, this remains to be seen, and indeed, retro-cue benefits have alternatively been attributed to a head-start retrieval explanation (Niklaus et al., 2019; Shepherdson, Oberauer, & Souza, 2018), whereby improved recognition of cued items reflects their retrieval into a focus of attention in preparation for the response phase. The important general point to be made here is that different methodologies should converge on a common structural model of working memory, with differences between them explicable in terms of alternative ways of deploying control processes to meet task demands.

Finally, we reflect that our investigation started from an interest in the concept of a multimodal episodic buffer specialized for storing bound representations. We have found evidence for a single-item focus of attention that serves the purpose of encoding and maintaining feature bindings in visual working memory. This is similar to Treisman’s original proposal, and builds on it. An obvious question is whether we should equate the focus of attention with the episodic buffer. There are at least two good reasons for being cautious. One is that we have yet to provide any evidence that the focus of attention revealed in experiments on visual working memory has the multimodal property of the episodic buffer. If so, there should be circumstances in which it is vulnerable to interference from a perceptual distractor in another modality, not just vision. It is known that an attended postdisplay auditory distractor disrupts memory for visual feature bindings (Zokaei, Heider, & Husain, 2014), but the crucial evidence concerns whether an unattended auditory distractor would do so, too, and whether the effect is specific to memory for the most recent item. A second reason for caution concerns capacity. The episodic buffer is assumed to hold chunks that can be quite large, spanning several items in the verbal domain, where memory for sentences benefits from linguistic knowledge in long-term memory (Allen, Hitch, & Baddeley, 2018; Baddeley, Hitch, & Allen, 2009). We think it likely that that the limited capacity of the focus of attention in our experiments reflects a limit on the scope for episodic integration due to the impoverished nature of the materials and their sequential presentation. Experiments with richer materials would be needed to shed light on whether the limit would be greater when, for example, a chess expert is required to remember the current state of a chess board.

As we noted earlier, the experimental program we have reviewed was initially motivated, at least in part, by Anne Treisman’s work on binding in perception and working memory. As such, we have continued to employ tasks measuring memory for binding between visual features (in particular, color and shape) throughout this series. We assume that the findings and their interpretations are not necessarily limited to this category of stimulus and should extend across a range of materials, but this, of course, remains to be seen. For the present, we are content to have shown the continuing influence of Anne Treisman’s work on perception on our efforts to investigate binding in working memory.

## Footnotes

1 In all the experiments in which we compared change detection for bindings with change detection for features, conditions were presented in separate blocks of trials. We are grateful to a reviewer for pointing out that a participant could fail to detect a change in binding not only because the color was incorrectly associated with the shape but also because either the shape or the color was forgotten. The reviewer suggested using performance on feature trials to estimate the probability of both shape and color being remembered. This is given by color (Hits – False Alarms) × shape (Hits – False Alarms). If binding (Hits – False Alarms) is less than this amount, the implication is that sometimes both features were in memory but not their correct binding. We did these calculations, to obtain a purer estimate of memory for bindings in our change detection experiments, and then reran the analyses of variance in which binding and feature memory were compared. The patterns of significance for the interactions and main effects of interest remained the same in each case.
